# Giant axonal neuropathy (GAN): cross-sectional data on phenotypes, genotypes, and proteomic signature from a German cohort

**DOI:** 10.1007/s00415-024-12744-z

**Published:** 2024-12-16

**Authors:** Andrea Gangfuß, Guido Goj, Silke Polz, Adela Della Marina, Andreas Hentschel, Katja Ahlbory, Timo Deba, Urania Kotzaeridou, Elisabeth Schuler, Astrid Pechmann, Uta Diebold, Gerhard Kurlemann, Lucas Heinzkyll, Dirk Schmitt, Kevin Rostasy, Tobias Ruck, Johann Böhm, Andreas Roos, Ulrike Schara-Schmidt

**Affiliations:** 1 Department of Pediatric Neurology, Centre for Neuromuscular Disorders, C-TNBS, University Duisburg-Essen, Essen, Germany; 2 Department of Pediatric Neurology, Children’s Hospital Datteln, University Witten/Herdecke, Datteln, Germany; 3 Rheinhessen-Fachklinik – Mainz, Mainz, Germany; 4 Leibniz-Institut für Analytische Wissenschaften – ISAS – e.V., Dortmund, Germany; 5 Kinderkrankenhaus Amsterdamer Straße, Sozialpädiatrisches Zentrum, Kliniken Köln, Cologne, Germany; 6 Universitätsklinikum Münster, Klinik Für Kinder- und Jugendmedizin, Allgemeine Pädiatrie, Bereich Neuropädiatrie, Münster, Germany; 7 Division of Pediatric Neurology and Metabolic Medicine, Department of Pediatrics I, Medical Faculty of Heidelberg, Heidelberg University, Heidelberg, Germany; 8 Department of Neuropediatrics and Muscle Disorders, Medical Center - University of Freiburg, Faculty of Medicine, University of Freiburg, Freiburg, Germany; 9 Sozialpädiatrisches Zentrum Hannover, AUF DER BULT, Hannover, Germany; 10 Bonifatius Hospital Lingen, Kinderklinik, Lingen, Germany; 11 Medical Affairs, ITFpharma GmbH, Munich, Germany; 12 Department of Neurology, Medical Faculty and University Hospital Düsseldorf, Heinrich Heine University, 40225 Düsseldorf, Germany; 13 IGBMC (Institut de Génétique et de Biologie Moléculaire et Cellulaire), Université de Strasbourg, Inserm U1258, CNRS UMR7104, Illkirch, France; 14 Brain and Mind Research Institute, Children’s Hospital of Eastern Ontario Research Institute, University of Ottawa, Ottawa, ON K1H 8L1 Canada

**Keywords:** Giant axonal neuropathy, GAN Gene, Gigaxonin, Proteomics, Neurodegenrative, Consanguinity

## Abstract

Giant axonal neuropathy (GAN) is a progressive neurodegenerative disease affecting the peripheral and central nervous system and is caused by bi-allelic variants in the *GAN* gene, leading to loss of functional gigaxonin protein. A treatment does not exist, but a first clinical trial using a gene therapy approach has recently been completed. Here, we conducted the first systematic study of GAN patients treated by German-speaking child neurologists. We collected clinical, genetic, and epidemiological data from a total of 15 patients representing one of the largest cohorts described thus far. Average age of patients was 11.7 years at inclusion. The most frequently reported symptoms (HPO coded) were gait disturbance and muscle weakness, abnormality of muscle size, and abnormal reflexes. In line with the frequency of homozygous variants, in five families, parents reported being at least distantly related. In 14 patients, diagnosis was confirmed by molecular genetic testing, revealing eight different *GAN* variants, four being reported as pathogenic in the literature. Proteomics of white blood cells derived from four patients was conducted to obtain unbiased insights into the underlying pathophysiology and revealed dysregulation of 111 proteins implicated in diverse biological processes. Of note, diverse of these proteins is known to be crucial for proper synaptic function and transmission and affection of intermediate filament organisation and proteolysis, which is in line with the known functions of gigaxonin.

**Supplementary Information:**

The online version contains supplementary material available at 10.1007/s00415-024-12744-z.

## Introduction

Giant axonal neuropathy (GAN, MIM #256850), first described in 1972, is a rare autosomal recessive disorder with childhood-onset, characterised by progressive neurodegeneration of both, the peripheral and central nervous system [1]. Thus far, this neurological condition has been reported in approximately 75 families worldwide [2]. Genotypic data in combination with detailed phenotypic descriptions have been recorded for more than 100 patients [3, 4].

In general, two distinct clinical presentations have been described: the classical early onset, progressive neurodegenerative phenotype and a later-onset, slower-progressing axonal Charcot-Marie-Tooth (CMT) disease-like phenotype [5]. Some authors also define an intermediate phenotype [6]. Classical GAN phenotype typically manifests in infancy with distal weakness which expands to proximal weakness accompanied by cerebellar ataxia leading to loss of unassisted independent ambulation (LOA) by on average 8–10 years of age [4, 6]. However, other authors report LOA at the age of 14–16 years [7]. In addition to muscle weakness and ataxia, patients may also develop bulbar symptoms in the further course of the disease and typically have frizzy hair as a phenotypic identifier (“gestalt”). Nerve conduction studies show nerve length–dependent, sensorimotor neuropathy [4, 7, 8]. Magnetic resonance imaging (MRI) shows high signals on T2-weighted sequences in the anterior and posterior periventricular regions as well as in the cerebellar white matter, sometimes referred to as leukoencephalopathy or leukodystrophy. GAN is typically fatal, often due to pulmonary complications, by the third decade of life [4].

GAN-associated phenotypes are caused by bi-allelic pathogenic variants in the gene *GAN* (MIM *605379, linked to chromosome 16q23.2), which encodes gigaxonin, a ubiquitin ligase adaptor protein, member of the cytoskeletal BTB/kelch repeat family presumably acting as a cytoskeletal component. Thus, gigaxonin regulates intermediate filament turnover in the central and peripheral nervous systems [9]. The absence of gigaxonin leads to the accumulation and deposit of intermediate filaments in cellular inclusions, resulting in axonal swelling and the occurrence of eponymous “giant axons” visible by electron microscopy [10].

The *GAN* gene was identified in the year 2000 by Bomont and colleagues [11], and to date, 66 pathogenic and 24 likely pathogenic variants are listed in ClinVar [12]. The reported pathogenic variants comprised evenly distributed missense, nonsense, and splice site mutations as well as small insertions and deletions. An obvious mutation hotspot does not exist, and there is no apparent correlation between genotype and phenotype. The disease cannot yet be cured, but a first clinical gene therapy trial in the US has recently been completed showing a possible benefit in motor function scores at specific vector doses in a 1 year follow-up [2].

Here, we conducted a cross-sectional study to collect demographic, genetic, and clinical data from GAN patients clinically followed by paediatric neurologists across German-speaking countries (DACH-region, Germany, Austria, Switzerland). Our aims were to describe the main clinical and genetic features, explore a potential genotype/phenotype correlation, and identify preclinical factors influencing the progression of the disease by performing additional proteomic analyses of white blood cells derived from four GAN patients.

## Materials and methods

### Study design

This study was designed as a cross-sectional study collecting data from patients with confirmed diagnosis of GAN. Therefore, we conducted a survey using the ESNEK (Survey of Rare Neurological Diseases in Childhood and Adolescence), with its study centre located in Göttingen, Germany. To recruit patients, the ESNEK team sends an e-mail to registered paediatricians specialised in neuropediatrics in Germany, Austria and Switzerland regarding a specific rare neurological disorder. Nine centres from Germany contributed datasets of ten families with a total 15 of GAN patients. Clinical, genetic, and epidemiological data of GAN patients were collected from the participating centres using a standardised case report form (CRF), available from the corresponding author upon reasonable request. The data were collected retrospectively from clinical assessments of patient visits in routine clinical care. We did not receive any feedback from participating clinicians from Austria and Switzerland regarding treated GAN patients. In addition to the clinical data, we were able to obtain proteomic data sets derived from white blood cells from four patients (see below).

### Patient cohort

The inclusion criteria for the study were: (1) genetically confirmed diagnosis of a GAN-related neurological phenotype by identification of a compound heterozygous or homozygous variants in the *GAN* gene, or nerve biopsy findings strongly suggestive of GAN; and (2) sufficient data and clinical records on disease onset and progression, organ involvement, and muscle function status at last clinical assessment.

We categorised the patients in two phenotypes: the classic, severe, neurodegenerative, and a milder CMT-like phenotype based on the following characteristics:

- Severe: LOA before 10 years of age, MRI abnormalities, and/or clinical abnormality of the central nervous system (e.g., ataxia, dysarthria, dysdiadochokinesis, etc.).
- Mild: Independent ambulation beyond 10 years of age; ability to run and/or jump after 5 years of age.

We defined independent ambulation as the ability to walk 10 m without any assistance, or orthotic devices and ambulation with assistance as the ability to walk 10 m using any kind of external support (e.g., furniture, orthotics).

### Proteomic analyses

Proteomic profiling on white blood cells (purified from 7.5 ml EDTA blood samples) derived from four GAN patients (1, 5, 6, and 10) and five controls and subsequent data analyses was carried out as described previously [13].

### Data availability

The data that support the findings of this study are available from the corresponding author, upon reasonable request. The mass spectrometry proteomics data have been deposited to the ProteomeXchange Consortium via the PRIDE [14] partner repository with the dataset identifier PXD053070.

## Results

### Demographic data

A total of 15 patients from ten families diagnosed with GAN were enrolled from nine German centres. Age of patients at time of study inclusion ranged from 5.7 to 20.0 years with a median age of 11.7 years (SD 4.2). 13 (86.7%) patients were under 18 years old at the time of study enrolment. Eight (53.3%) were male and seven (46.7%) female. Eight (53.3%) patients originated from Syria, two (13.3%) from Turkey and from Germany, respectively. One patient (6.7%) was from Saudi Arabia, and in two (13.3%) patients, the origin was unknown. In 5/10 (50%) families, parents reported being at least distantly related. Table 1 gives an overview of the demographic, genetic and clinical data of our cohort.

**Table 1:** Overview of the described GAN cohort

Family	Patient	Variant (n/r)	Interpretation	ACMG criteria	Consan-guinity	Country of origin	Sex	Age at exam/age at death (years)	Ambulation status/loss of ambulation (years)	Central nervous system signs
1	1	c.1703C>A; p.Thr568Lys^n^	Missense mutation	Likely pathogenic	Unknown	Germany	m	11.3	8.0	Ataxia, dysarthria, dysdiadochokinesis, tremor, dysmetria
2	2	c.305T>C; p.Ile102Thr^r^	Missense mutation	Pathogenic	Yes	Syria	m	5.7	Ambulant	None
	3						m	11.0	Part-time wheelchair dependent	None
	4						m	12.3	Able to walk with orthotics	None
3	5	c.1502+1G>T; p.del459-501^r^	Splice site mutation	Pathogenic	Yes	Syria	f	18.6	10.0	Dysarthria, dysdiadochokinesis, dysmetria
	6						f	5.5	Ambulant	None
4	7	c.806G>A; p.Arg269Gln^r^	Missense mutation	Pathogenic	No	Germany	f	12.2	Able to walk with orthotics	None
5	8	c.1502+1G>T; p.del459-501^r^	Splice site mutation	Pathogenic	Yes	Syria	f	7.9	Ambulant	Ataxia
	9						f	9.0	Able to walk with orthotics	Ataxia
6	10	c.218T>C; p.Ile73Thr^n^	Missense mutation	VUS	No	Turkey	m	16.2/17.0	11.0	Dysarthria, dysdiadochokinesis
	11						f	13.9/20.0	Full-time wheelchair dependent	Dysdiadochokinesis
7	12	c.1102G>A; p.Gly368Arg^r^	Missense mutation	Pathogenic	Unknown	Unkown	f	7.3	Ambulant	None
8	13	c.998_1006del; p.Gly333_Asp335del^n^	In-frame deletion	VUS	Yes	Syria	m	12.3	Ambulant	Dysarthria, dysdiadochokinesis
9	14	c.1006G>T; p.Glu336*^n^	Nonsense mutation	Pathogenic	Yes	Saudi Arabia	m	11.9	Ambulant	None
10	15	NA	NA	NA	No	Unkown	m	14.0/14.3	11.0	None

*ACMG* American College of Medical Genetics, *f* female, *m* male, *n* novel variant, *NA* not applicable, *r* reported variant, *UNK* unknown

### Clinical characteristics

From the 15 patients described here, 13 (86.7%) had a classical and one a mild CMT-like phenotype. Owing to the young age, a final classification was not possible for a single patient. Abnormal gait was mentioned in all patients as one of the first symptoms, muscle weakness was notable in 13 (86.7%) patients, and muscle hypotonia in 7 (46.7%). Gait abnormalities were noted at a median age of 2.0 years (SD 1.3). At time of the last follow-up, 6 (40%) patients were able to walk >10 m independently and 9 (60%) were not able to walk 10 m without assistance, including 3 (20%) using orthotic aids, 2 (13.3%) being part-time wheelchair dependent and 4 (26.7%) being full-time wheelchair dependent. LOA occurred between 8.8 and 11 years with a median age of 10.5 years (SD 0.9). Further clinical characteristics of our cohort are summarised in Table 2.

**Table 2:** Clinical characteristics of the described GAN cohort, listed according to the selected Human phenotype ontology (HPO) terms

Human phenotype ontology terms	*n* = 15 (%)
Development	
Delayed gross motor development	4 (26.7)
Delayed fine motor development	2 (13.3)
Delayed ability to walk	3 (20.0)
Delayed speech and language development	2 (13.3)
Intellectual disability	4 (26.7)
Gait	
Gait disturbance	15 (100.0)
Gait ataxia	5 (33.3)
Frequent falls	10 (66.7)
Steppage gait	6 (40.0)
Muscle	
Muscle weakness	15 (100.0)
Generalised muscle weakness	5 (33.3)
Axial muscle weakness	6 (40.0)
Distal muscle weakness	12 (80.0)
Fatigable weakness	5 (33.3)
Weakness of facial musculature	4 (26.7)
Muscular hypotonia	14 (93.3)
Abnormality of muscle size	12 (80.0)
Generalised amyotrophy	7 (46.07)
Proximal upper limb amyotrophy	4 (26.07)
Proximal lower limb amyotrophy	4 (26.07)
Distal upper limb amyotrophy	5 (33.03)
Distal lower limb amyotrophy	8 (53.03)
Neurologic	
Abnormal reflexes	12 (80.0)
Areflexia	11 (73.3)
Pyramidal sign	2 (13.3)
Abnormality of coordination	7 (46.7)
Slurred speech	4 (26.7)
Dysdiadochokinesis	5 (33.3)
Truncal ataxia	3 (20.0)
Seizures	2 (13.3)
Abnormality of the eye	9 (60.0)
Nystagmus	6 (40.0)
Bone	
Scoliosis	7 (46.7)
Abnormality of joint mobility	8 (53.3)
Limb joint contracture	8 (53.3)
Ankle flexion contracture	7 (46.7)
Abnormal foot morphology	8 (53.3)
Talipes	4 (26.7)
Pes planus	7 (46.7)
Pes excavatus/Pes cavus	2 (13.3)
Abnormality of the hand	4 (26.7)
Phenotype	
Phenotypic abnormality	8 (53.3)
Curly/Frizzy hair	14 (93.3)
Abnormal skull morphology	2 (13.3)
Respiratory	
Abnormality of the respiratory system	8 (53.3)
Sleep apnoea	3 (20.0)
Need for ventilatory support	2 (13.3)
Diverse	
Abnormal autonomic nervous system physiology	7 (46.7)
Abnormal digestive system physiology	6 (40.0)
Feeding difficulties	4 (26.7)
Abnormality of the endocrine system	6 (40.0)
Abnormal autonomic nervous system physiology	7 (46.7)
Autonomic bladder dysfunction	4 (26.7)
Premature death	3 (20.0)

**Fig. 1:**
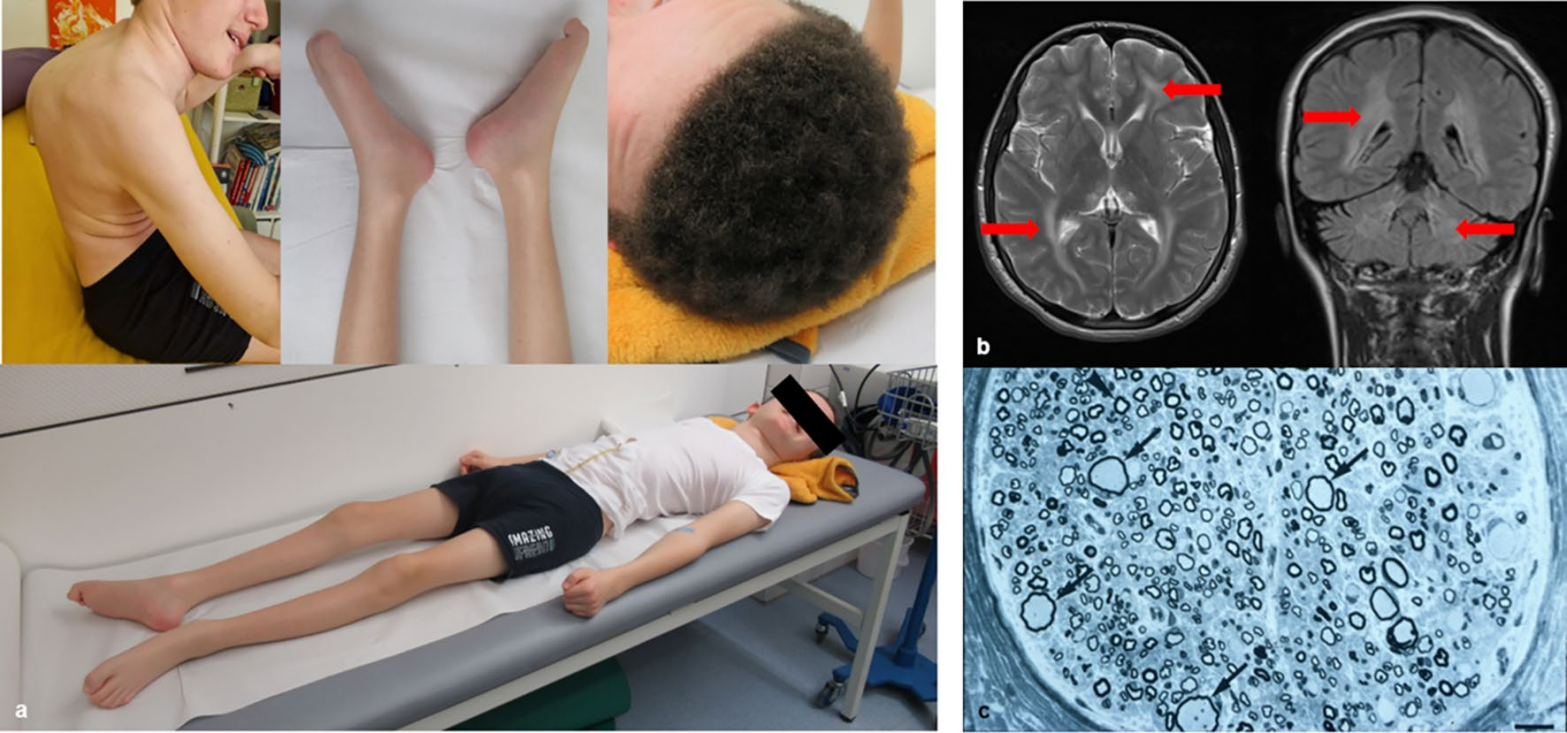
Clinical data of GAN patients. **A** Patient 1 at 12 years of age showing typical clinical signs for GAN, e.g., kyphoscoliosis, pes planus, generalised amyotrophy, contractures of interphalangeal joints and frizzy hair, as well as being fully wheelchair dependent at the time of examination due to profound muscle weakness. **B** T2 axial (left) and T1 coronar (right) MRI of patient 5 at 15 years of age showing typical diffuse signal hyperintensities of the white matter of the cerebrum and cerebellum. **C** Electron microscopy of suralis nerve biopsy of patient 15 showing enlarged axons (indicated by arrows) surrounded by relatively thin myelin sheaths in contrast to normal axons (indicated by arrowheads)

Figure 1 shows the classical GAN phenotype in patient 1 including kyphoscoliosis, pes planus, generalised amyotrophy, contractures of interphalangeal joints and frizzy hair, as well as being fully wheelchair dependent at the time of examination due to profound muscle weakness. For patient 5 cranial MRI was available showing typical diffuse signal hyperintensities of the white matter of the cerebrum and cerebellum, especially periventricularly, in the area of both thalami, the midbrain and the pons. Sural nerve biopsy was performed in patient 15 presenting the typical enlarged axons at the ultra-structural level (Fig. 1).

### Genetic characteristics

**Fig. 2:**
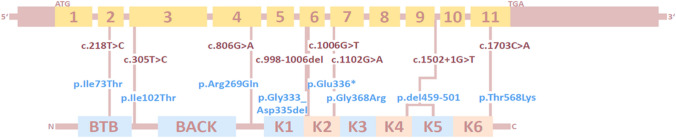
Genetic data of GAN patients. All *GAN* variants included in this study arranged by cDNA (dark red) and protein (blue) position. All identified variants are homozygous. Gene sequence and protein domains were obtained from the ensemble and NCBI databases

Exome, panel, or direct gene sequencing detected pathogenic *GAN* variants (Fig. 2) in nine families in the present study. All variants were homozygous, and the segregation was verified by Sanger sequencing on DNA samples from the available family members.

The pathogenic variants encompassed five missense mutations (c.218T>C, p.Ile73Thr; c.305T>C, p.Ile102Thr; c.806G>A, p.Arg269Gln; c.1102G>A, p.Gly368Arg; c.1703C>A, p.Thr568Lys), an in-frame deletion of 9 nucleotides (c.998-1006del, p.Gly333_Asp335del), an essential splice site mutation leading to the exclusion of the in-frame exon 9 (c.1502+1G>T, p.del459-501), and a single nonsense mutation predicted to involve mRNA decay (c.1006G>T, p.Glu336*). The predominance of missense mutations confirms previous studies on giant axonal neuropathy [15]. Only exon 6 harboured two pathogenic variants (c.998-1006del and c.1006G>T), and the other mutations were evenly distributed over the gene and were localised in exons 2, 3, 4, 7, and 11.

The five missense mutations and both in-frame deletions affected conserved amino acids in the BTB domain (c.218T>C), in Kelch domains 2 (c.1102G>A and c.998-1006del), 4 and 5 (c.1502+1G>T), or 6 (c.1703C>A), or in linker regions without known function (c.305T>C and c.806G>A), emphasising the absence of a mutation hotspot and suggesting that any modification of the peptide sequence is pathogenic. This is supported by the almost complete absence of homozygous variants in the coding regions of *GAN* in the gnomAD population database.

Of note, the c.1502+1G>T splice site mutation was found in two unrelated families in our cohort. However, both families originate from Syria, indicating a founder effect and a distant family relationship. Four of the eight different *GAN* mutations were described in previously reported families: c.305T>C [16]; c.806G>A [4, 17], c.1102G>A [17], and c.1502+1G>T [18] and all four are classified as pathogenic. Following the ACMG criteria and the presence of other homozygous nonsense mutations in reported *GAN* patients, the c.1006G>T nonsense mutation is classified as pathogenic. The impact and implication of the c.1703C>A missense mutation was supported by additional hair analysis showing altered keratin structure typical for GAN and is classified as likely pathogenic. Both remaining variants (c.218C>T and c.998-1006del) are classified as VUS (variants of uncertain significance). Whilst the c.218C>T missense variant is listed once at the heterozygous state with a CADD score of 27.1, the c.998-1006del deletion is absent from the gnomAD database. Together with the confirmed segregation in the affected families, the high degree of conservation of the *GAN* gene, and the occurrence of similar mutations in other *GAN* patients in combination with very suggestive phenotype, this strongly suggests a pathogenic effect of the identified mutations.

### Proteomic analyses

Blood samples from patients 1, 5, 6, and 10 presenting with severe phenotypes were available to isolate white blood cells and to perform proteomic analyses to obtain unbiased insights into the cellular pathophysiology. The utilisation of white blood cells to this end was also driven by results of our previous studies highlighting that this cellular population is suitable to investigate molecular processes underlying in inherited neuropathies [13, 19, 20].

**Fig. 3:**
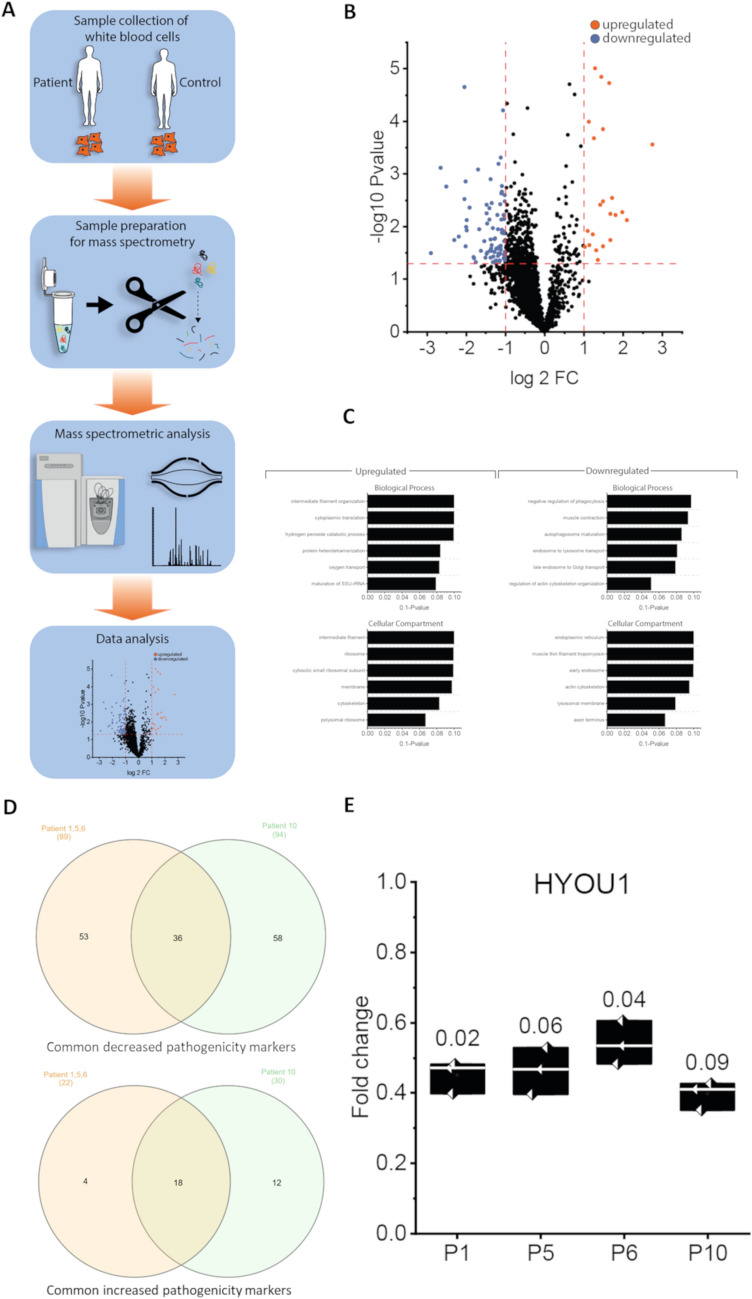
Proteomic data of GAN patients. **A** Schematic representation of the proteomic workflow applied on patient-derived white blood cells. **B** Volcano plot depicting and highlighting the significant upregulated proteins (red; FC ≥ 2) and downregulated proteins (blue; FC ≤ 0.5). **C** GO-Term analysis; bar graphs depicting upregulated and downregulated biological processes in regard to the significant upregulated and downregulated proteins. **D** Comparison of proteomic signatures of patients 1, 5, and 6 (grouped) versus 10 to pinpoint proteins serving as pathogenicity markers unveils 54 proteins (36 decreased and 18 increased) commonly dysregulated in GAN patients. **E** Box plot of HYOU1 abundances in white blood cells of patients 1, 5, 6, and 10 showing a decrease of this co-chaperone in all patients. Numbers above boxes display the respective statistical significance (p-ANOVA) of HYOU1-decrease in the individual patient (P) sample

Before, gigaxonin abundance in white blood cells was confirmed based on an in-house generated spectral library revealing the presence of four unique tryptic peptides for gigaxonin in human white blood cells (Fig. 3), thus for the first time unveiling the presence of this neurological relevant protein in white blood cells and declaring same ones as a suitable in vitro system to investigate molecular consequences of loss of functional gigaxonin.

Indeed, our mass spectrometry-based approach defined a proteomic signature characterised by the significant dysregulation of 111 proteins (DAPs = differentially abundant proteins), whereby 22 are increased and 89 are decreased, respectively. A GO-term based in silico study was next conducted to pinpoint biological processes and cellular compartments affected by the respective protein dysregulations. Increased DAPs impact on different biological processes, including intermediate filament organisation, cytoplasmic translation, hydrogen peroxide catabolic processes along with oxygen transport, protein heterotetramerization, as well as maturation of SSU-rRNA. Instead, decreased proteins impact on muscle contraction, regulation of phagocytosis, autophagosome maturation along with proper endosome to lysosome transport and late endosome to Golgi transport, as well as regulation of actin cytoskeleton organisation. Cellular compartments affected by increased proteins include cytoskeleton including intermediate filaments, ribosomes, and cellular membranes. Cellular compartments affected by decreased proteins also include cytoskeletal proteins (muscle thin filament tropomyosin and actin), the endoplasmic reticulum, endosomes, the lysosome membrane, and of note the axon terminus. Especially, in the context of predicted vulnerability of axon terminus, it is important to note that diverse proteins with known functions in synaptic function are decreased including neurogranin, LRP chaperone MESD, alpha-synuclein, and synaptogyrin-2 (Suppl. Tab. 1). GAN was not detected in our proteomic profiling approach.

Moreover, we used our proteomic profile data to evaluate the pathogenicity of the novel variant identified in patient 10: a comparison of the individual proteomic signature of this patient with the patient harbouring known pathogenic variants revealed the common decrease of 36 and the common increase of 18 proteins (Suppl. Tab. 2) declaring these once to potential pathogenicity markers. Of note, some of these proteins, such as tropomyosins, alpha-synuclein, and neurogranin, are known to play diverse functions in proper neuromuscular transmission. Given that the results of the global profiling of all patients versus healthy controls unveiled a decrease of HYOU1 (GRP170) (Suppl. Tab. 1), a chaperone with crucial functions in neurodegeneration, we addressed the suitability of HYOU1 to also serve as a marker protein allowing to evaluate the pathogenicity of novel genetic GAN variants. Indeed, although not statistically significant in all patient-derived samples, HYOUI is constantly decreased (Fig. 3E).

## Discussion

Here, we report demographical, clinical, and molecular genetic data of 15 patients from 10 families living in Germany with confirmed giant axonal neuropathy. Four of the eight identified *GAN* mutations were described in previously reported families. According to ACMG criteria, one of the remaining mutations was classified as pathogenic, one as likely pathogenic, and two as VUS. The survey revealed exclusively homozygous variants, and accordingly, consanguinity (defined as being at least distantly related) was reported by half of the families.

Based on the analysis of the clinical and genetic data of our patients, a genotype–phenotype correlation can only be partially drawn. From the 15 patients described here, 13 presented with a classical GAN phenotype, one patient showed mild manifestations, and one patient was too young for a definite classification. The higher prevalence of GAN cases with classical clinical presentation is in agreement with the previous studies [4].

The patient with the mild phenotype carried the homozygous c.806G>A (p.Arg269Gln) mutation and presented with GAN-typical early childhood-onset hypotonia, muscle weakness, contractures, skeletal anomalies, and abnormal gait without clinical central nervous system involvement. Brain MRI was unremarkable, and the patient was still able to walk with orthotics at the age of 12, contrasting the more severe cases in our cohort. The same pathogenic p.Arg269Gln variant was previously described in an unrelated family, and both affected members manifested a similar phenotype as our patient with normal white matter on MRI and loss of ambulation beyond the age of 12 [17]. Both patients also presented with ataxia, which was absent in our patient. However, the age of the previously reported patients is not specified, and it is possible that first signs of ataxia may occur in later disease stages in milder GAN cases. Another patient with the p.Arg269Gln variant was 6 years old at the last clinical examination [4] and does not permit any comparison with our patient. Importantly, the differentiation between classical and mild GAN phenotypes was previously solely based on motor parameters [4].

To broaden the classification criteria and evaluate a more systemic aspect of the disease, we also included CNS symptoms and cranial MRI findings, which may also be used as a standard for describing the severity of GAN cases in the future. The absence of kinky hair as an indicator of a mild phenotype cannot be used as classification criterion as shown by CNS signs indicating classical GAN in our patient with the in-frame deletion of 9 nucleotides (c.998-1006del).

As a common rule, nonsense mutations are rather associated with a classical GAN phenotype, whilst missense mutations are found in milder cases [4]. This is also confirmed by our cohort and the presence of nystagmus and leukoencephalopathy evidencing a CNS involvement in the patient with the c.1006G>T (p.Glu336*) nonsense mutation. However, the patient was still ambulant at the age 12, demonstrating that distinct clinical signs of classical and mild giant axonal neuropathy can sometimes overlap, and suggesting a similar loss-of-function mechanism for GAN missense and nonsense mutations.

From a general point of view, a longitudinal study on a well-characterised patient cohort may be more adapted for the establishment of a genotype/phenotype correlation than a cross-sectional study. Longitudinal studies also provide the possibility to apply standardised clinical examinations with validated scales as the CMT Neuropathy Exam Scores (CMTNS/CMTES, CMTPedS/CMTInfS) or the Motor Function Measurement (MFM), although they often appear as rather complex for routine clinical practise but are absolutely needed as outcome measures for clinical studies.

In a phase 1 gene therapy trial conducted by Bharucha-Goebel and colleagues, four different doses of intrathecal AAV vector with a gigaxonin-encoding transgene (scAAV9/JeT-GAN) were administered to 14 GAN patients with classical, severe phenotype. Overall, specific vector doses seemed to improve motor function scores and sensory-nerve action potential curves in single patients, but serious adverse events were also noted [2]. Accordingly, further studies are needed to determine the safety and efficacy of intrathecal AAV-mediated gene therapy in GAN.

In view of further clinical trials for GAN, we aimed to identify suitable biochemical sensors reflecting disease progression and the efficiency of therapeutic approaches. For the first time, we provide a catalogue of proteins affected by loss of functional gigaxonin in leukocytes. Given that gigaxonin is itself abundant in white blood cells as outlined based on our spectral library data, the fold of restoration of DAPs should ideally be correlated with the achieved level of expression of exogenous *GAN* within these cells. From our perspective, this approach is especially grounded on the observation that dysregulated proteins are in line with the known function of gigaxonin as a cytoskeletal component that directly or indirectly plays an important role in neurofilament architecture and moreover acts as a substrate-specific adapter of an E3 ubiquitin-protein ligase complex which mediates the ubiquitination and subsequent proteasomal degradation of target proteins. Results of our GO-term-based pathway analyses clearly indicate affection of cytoskeleton whereby increased proteins impact on neurofilament organisation. In addition, our proteomic data hint towards perturbed protein clearance based on the decreased DAPs. This biochemical finding is accompanied by decreased level of a variety of proteins involved in vesicular transport, such as sorting nexins, SNARE-associated proteins, sortilin, and vesicle-associated membrane protein 8 and dynactin subunit 2 amongst others. Of note, DCTN2 is assumed to play a role in synapse formation during brain development and a genetic study introduced *DCTN2* as a candidate gene for intermediate Charcot–Marie–Tooth disease in a Norwegian family [21]. Since the maturation of the autophagosome is influenced by regular vesicle transport, the identified protein dysregulations might impact on the function of the proteasome, which is presumably already altered by the loss of functional gigaxonin and cannot be compensated efficiently enough by autophagy. Of course, further functional studies are required to validate this hypothesis and to obtain more insights into the complex pathobiochemistry.

Nevertheless, proper vesicle transport is required for efficient synaptic transmission and results of our proteomic profiling approach showed reduced abundance of proteins playing crucial roles in synaptic transmission, such as neurogranin [22], LRP chaperone MESD (http://www.uniprot.org/uniprotkb/Q14696/entry), and synaptogyrin-2 (http://www.uniprot.org/uniprotkb/O43760/entry).

In the context of ataxia as a leading symptom of GAN-related neurodegeneration, it is important to note that HYOU1 (GRP170/ ORP150), a co-chaperone of BiP (the major chaperone of the endoplasmic reticulum), is decreased in white blood cells derived from GAN patients and that bi-allelic variants in three other binding partners of BiP (SIL1, INPP5K, and DNAJC3) were already linked to phenotypes associated with ataxia [23–25], thus indicating a profound role of BiP and its binding partners in cerebellar maintenance. In accordance with our finding of decreased HYOU1 in white blood cells derived from GAN patients, Zhao and colleagues in 2010 reported that overexpression of HYOU1 prevents ER stress and rescues neurodegeneration in Sil1(-/-) mice as an in vivo model of cerebellar ataxia [26, 27], whereas decreasing expression of HYOU1 exacerbates this phenotype [28]. Moreover, deficiency of the alternative BiP co-chaperone SIL1 causes degenerative changes of axons in mice and human [29]. In the light of these facts, one might assume that HYOU1 might represent a promising molecular target to ameliorate GAN-related neurodegeneration. Taking the above-mentioned protein dysregulations of presumed clinical relevance as well as the lack of a convincing genotype-phenotype correlation into consideration, biochemical studies on white blood cells derived from mildly versus severely affected GAN patients might allow to identify modifying proteins. However, unfortunately, no biomaterial derived from mildly affected patients was available for this study.

To concrete, these data on the natural history of the disease as well as the catalogue of proteins that may serve as minimally invasive but cellular biomarkers (e.g., HYOU1) are not only important to evaluate the pathogenicity of genetic variants of unknown significance but might also serve as biochemical read-out measures for future gene therapy trials. However, further studies on larger cohorts are needed to finally evaluate the potential of these white blood cell-related proteins to serve as suitable GAN biomarkers.

## Supplementary Information

Below is the link to the electronic supplementary material.

Supplemental Table 1: Table of significantly dysregulated proteins unveiled by the global comparison of GAN patients 1, 5, 6 and 10 versus healthy controls. (DOCX 12 kb)

Supplemental Table 2: Table of proteins dysregulated in patient 10 compared to grouped patients 1, 5, and 6 to decipher proteins holding the potential to serve as minimal-invasive pathogenicity markers. (XLSX 10 kb)

## Declarations

### Reviewer information

Reviewer can access the proteomic dataset by logging in to the PRIDE website using the following account details: Username: reviewer_pxd053070@ebi.ac.uk. Password: qVhgNbq4WUBg.
