# Supplementary material for: Giant axonal neuropathy (GAN): cross-sectional data on phenotypes, genotypes, and proteomic signature from a German cohort
Source: J Neurol. 2024 Dec 16;272(1):63. doi: 10.1007/s00415-024-12744-z (PMC11649756; doi:10.1007/s00415-024-12744-z)
Supplement: Supplementary file 1 — Supplemental Table 1: Table of significantly dysregulated proteins unveiled by the global comparison of GAN patients 1, 5, 6 and 10 versus healthy controls. (DOCX 12 kb) [file 415_2024_12744_MOESM1_ESM.docx]

| **Accession** | **Gene** | **Protein Description** | **Unique Peptides** | **Patient/Control** | **pValue** |
| --- | --- | --- | --- | --- | --- |
| P37840 | SNCA | Alpha-synuclein | 7 | 0.49 | 0.014 |
| O43760 | SYNGR2 | Synaptogyrin-2 | 4 | 0.46 | 0.028 |
| Q14696 | MESD | LRP chaperone MESD | 2 | 0.43 | 0.045 |
| Q92686 | NRGN | Neurogranin | 2 | 0.24 | 0.000 |
